# The Perturbation of Infant Gut Microbiota Caused by Cesarean Delivery Is Partially Restored by Exclusive Breastfeeding

**DOI:** 10.3389/fmicb.2019.00598

**Published:** 2019-03-26

**Authors:** Yu Liu, Shengtang Qin, Yilin Song, Ye Feng, Na Lv, Yong Xue, Fei Liu, Shuxian Wang, Baoli Zhu, Jingmei Ma, Huixia Yang

**Affiliations:** ^1^Department of Obstetrics and Gynecology, Peking University First Hospital, Beijing, China; ^2^Beijing Key Laboratory of Maternal Fetal Medicine of Gestational Diabetes Mellitus, Beijing, China; ^3^Key Laboratory of Pathogenic Microbiology and Immunology, Institute of Microbiology, Chinese Academy of Sciences, Beijing, China; ^4^Beijing Key Laboratory of Antimicrobial Resistance and Pathogen Genomics, Beijing, China; ^5^Department of Pathogenic Biology, School of Basic Medical Sciences, Southwest Medical University, Luzhou, China

**Keywords:** infant, early life, gut microbiome, delivery mode, breastfeeding, postnatal antibiotic exposure

## Abstract

Early establishment of the infant gut microbiome has been attributed to various environmental factors that may influence long-term health. The aim of this study was to determine the single and combined impacts of the delivery mode, feeding pattern and postnatal antibiotic exposure on the initial establishment of infant gut microbiome at 6 weeks postpartum. A cross-sectional study was conducted at a single center in China. Fecal samples were collected from 120 infants at 6 weeks postpartum. The V3-V4 regions of 16S rRNA gene were analyzed by Illumina sequencing, and clinical information was obtained from medical records and questionnaire survey. Compared with vaginally delivered infants, the gut microbial community structure of cesarean delivered infants were significantly different (*P* = 0.044), in parallel with the decreased relative abundance of *Bifidobacterium* (*P* = 0.028), which contrasts with the normal gut microbial establishment. Using the vaginally delivered and exclusively breastfed (VB) infants as a reference, the comparative analysis of cesarean delivered and exclusively breastfed (CB) infants with cesarean delivered and mixed-fed (CM) infants showed that both within- and between-group UniFrac distance were significantly smaller in CB infants (*P* < 0.001, *P* < 0.001). LEfSe analysis showed that the relative abundances of *Enterococcus, Veillonella*, and *Faecalibacterium* were significantly different between CB and CM infants, whereas the relative abundances of those genera in VB infants were close to those of CB infants, and distinct from those of CM infants. Additionally, no significant difference of microbial composition, alpha diversity, or community structure was observed between postnatal antibiotics exposed infants and unexposed infants. In summary, delivery mode had a significant impact on the infant gut microbial community structure and composition, and the gut microbiota was disturbed in infants delivered by cesarean section. However, our study showed that this disturbance of gut microbiota in cesarean delivered infants was partially restored by exclusive breastfeeding in comparison with mixed feeding. No distinct impact of postnatal antibiotic exposure on infant gut microbiome was found at 6 weeks of age.

## Introduction

The gut microbiota plays a vital role in intestinal barrier function, metabolic reaction and trophic structure (Matamoros et al., 2013), and the colonization and maturation of gut microbiota in early human life are processes closely associated with infant growth, nutritional and immunological development (Round and Mazmanian, 2009). A gut microbiota that is not well established is associated with metabolic and allergic diseases, such as diarrhea and malnutrition in Bangladeshi children (Subramanian et al., 2014), overweight and obesity at 7 years old (Kalliomaki et al., 2008), diabetes mellitus (Qin et al., 2012), infant asthma during the first 100 days of life (Arrieta et al., 2015), and pediatric Crohn’s disease (Gevers et al., 2014). Although recent studies have reported that microbiome acquisition begins in utero (DiGiulio et al., 2008; Jimenez et al., 2008; Gosalbes et al., 2013; Aagaard et al., 2014; Blaser and Dominguez-Bello, 2016; Collado et al., 2016; Zhu et al., 2018), those evidences are extremely weak (Perez-Munoz et al., 2017). Most evidence suggests that bacterial colonization begins at birth and is predominated by facultative anaerobes, such as *Enterococcus* and *Staphylococcus*, in healthy infants during first few weeks where after obligate anaerobes, such as *Bacteroides, Bifidobacterium*, and *Clostridium*, and the obligate/facultative ratio increases with age (Dogra et al., 2015; Nagpal et al., 2017). Despite the interindividual variations, Firmicutes, Bacteroidetes, Actinobacteria, and Proteobacteria are the most dominant phyla, while *Bacteroides, Bifidobacterium, Clostridium, Prevotella*, Enterobacteriaceae, and *Enterococcus* are the major bacterial groups that are involved in initial colonization of intestinal microbiota (Nagpal et al., 2017). Gradually, the infants gut microbiota shows a greater diversity and a more complex structure, acquiring a mature gastrointestinal (GI) microbiota resembled adults by around the 3 years of age (Yatsunenko et al., 2012; Tamburini et al., 2016).

Initial colonization of the intestinal microbiota is influenced by various prenatal and postnatal factors (Tamburini et al., 2016). Among those environmental factors, delivery mode (Dominguez-Bello et al., 2010, 2016), feeding pattern (Backhed et al., 2015; Madan et al., 2016) and antibiotic usage (Yassour et al., 2016; Nogacka et al., 2017) are major determinants. Vaginally delivered infants are initially colonized by *Lactobacillus*, a predominant genus in the maternal vaginal canal, whereas cesarean delivered infants are initially colonized by maternal skin bacteria such as *Staphylococcus* and *Streptococcus* (Dominguez-Bello et al., 2010, 2016), and this transient difference appears to be corrected after weaning (Rutayisire et al., 2016). Cesarean delivery is considered to be associated with a perturbation and delayed maturation of gut microbiota in early life, which may in turn be associated with increased risk of childhood obesity (Blustein et al., 2013), asthma (Kolokotroni et al., 2012) and immune diseases (Sevelsted et al., 2015). However, no study has elucidated the causality or mechanism of this association, which may be due to the lack of “bacterial baptism” of vaginal birth or due to confounding factors associated with cesarean delivery, such as maternal obesity, antibiotic administration, gestational age and breastfeeding pattern (Stinson et al., 2018).

Conversely, breastfeeding is associated with a lower risk of allergic diseases (Wopereis et al., 2018) and obesity (Forbes et al., 2018; Mueller and Blaser, 2018) than formula feeding. Exclusively breastfed infants harbor more *Bifidobacterium* and *Lactobacillus* than formula-fed infants, and those two anaerobia bacteria result in a more acidic environment in the GI tract that defends against common pathogens. Furthermore, antibiotic exposure is also linked to increased risks of diabetes (Kilkkinen et al., 2006) and obesity (Azad et al., 2014). Both prenatal and postnatal antibiotic exposure are thought to disrupt the infant gut microbiome that lead to loss of beneficial bacteria and attenuation of microbial diversity (Bokulich et al., 2016). However, the alteration of microbial diversity is relatively modest after the first year of life (Yassour et al., 2016).

Altogether, previous studies regarding delivery mode, feeding pattern and antibiotic exposure have shown some disparities in the initial colonization of infant gut microbiota (Rutayisire et al., 2016), but few studies have explored the independent and combined impacts of delivery mode, feeding pattern and postnatal antibiotic exposure following adjustment for each variable. Hence, we conducted a cross-sectional study in a single geographical area (Beijing, China) to identify the independent and combined impacts of delivery mode (vaginally or cesarean delivery), feeding pattern (exclusive breastfeeding or mixed feeding) and postnatal antibiotic exposure (yes or no) on the gut microbiota of 120 Chinese infants at 6 weeks postpartum, as well as explore the restorative effect of exclusive breastfeeding on gut microbiome of cesarean delivered infants.

## Materials and Methods

### Study Participants and Clinical Information

This cross-sectional study was conducted at a single center in China and included 120 infants. Six weeks was chosen for sample collection because the feeding pattern is well established at this stage and it corresponds to a routine time for the first maternal and infant postpartum visit in China, which largely facilitated sample collection. Clinical information for the 120 infants was obtained from electronic medical records and questionnaire surveys (Supplementary Table S1). Considering the difference in the offspring microbiome delivering by labored cesarean section and unlabored cesarean section (Azad et al., 2013; Chu et al., 2017), and the maternal pregnancy complication may also exert an influence on infant microbiota (Stanislawski et al., 2017; Wang et al., 2018), we collected the information of the type (labored or elective) and cause of cesarean section (Supplementary Table S1). Exclusive breastfeeding was defined as infants who were fed breast milk exclusively after birth. Mixed feeding was identified as a mixture of varying proportions of breast milk and formula milk. Detailed information regarding antibiotic use, including the indication, type, dosage, duration, and interval time to sample collection, was recorded (Supplementary Table S2). This project was approved by the Ethics Committee of Peking University First Hospital (V2.0/201504.20), and informed consent was obtained from all participants.

### Sample Collection, DNA Extraction, and Sequencing

Infant fecal samples were collected at approximately 6 weeks postpartum. Fresh fecal samples were placed into sterile tubes filled with RNA store later (Tiangen, China, DP408-02) and frozen at -80°C within 2 h. DNA was extracted with the QIAamp PowerFecal DNA kit (Qiagen, Hilden, Germany) following the manufacturer’s protocols, and the V3-V4 region of the 16S rRNA gene was amplified by polymerase chain reaction (PCR) with the 341 forward primer (5′ CCTACGGGNBGCASCAG) and 805 reverse primer (5′ GACTACNVGGGTATCTAATCC). The PCR reactions were performed in 25 μL volume with 1 U HiFi HotStart DNA Polymerase (KK2502, Kapa Biosystems), 12.5 ng template DNA and 5 μM forward and reverse primers with the following amplification program: initial denaturation at 95°C for 3 min; 25 cycles of denaturation at 95°C for 30 s, annealing at 55°C for 30 s and extension at 72°C for 30 s; final extension at 72°C for 5 min and hold at 4°C. The amplicons were cleaned using the AMPure XP beads (A63882, Beackman) to purify the 16S V3 and V4 amplicons away from the free primers and primer dimers according to the manufacturer’s instructions. The PCR products were sequenced on the Illumina HiSeq 2500 platform, and Fast Length Adjustment of Short Reads (FLASH) was used to merge paired-end reads from sequencing (Magoc and Salzberg, 2011). Low-quality reads were filtered with the FASTQ quality filter (-p 90 -q 25 -Q33) using the FASTX Toolkit 0.0.14, and chimeric reads were removed by USEARCH 64-bit v8.0.1517. The number of reads for each sample was normalized based on the smallest sample size by random subtraction. OTUs (operational taxonomic units) were aligned by the UCLUST algorithm with 97% identity and taxonomically classified using the SILVA 16S rRNA database v128. Alpha and beta diversity were evaluated using Quantitative Insights Into Microbial Ecology (QIIME) and calculated based on weighted and unweighted UniFrac distance matrices. The detailed protocol is shown on the website^1^.

### Statistical Analysis

The microbial alpha diversity (within individual) was evaluated with two indexes: the OTU number and Shannon index. The beta diversity (between individuals) was visualized by two-dimensional principal coordinates analysis (PCoA) of weighted and unweighted UniFrac distance matrices, in 1000 permutations, and statistical comparisons of groups were performed with the non-parametric MANOVA methods using the Adonis function in vegan R package. To identify distinct taxonomic bacterial biomarkers, the linear discriminant analysis effect size (LefSE) was determined by the LDA log score (cut-off ≥3) (Segata et al., 2011). Comparisons between groups were analyzed with parametric (Student’s *t*-test) or non-parametric tests (Mann-Whitney *U*-test). We assigned significance as *P* < 0.05.

## Results

### Cohort Characteristics

The 120 infants were divided into antibiotics group (A group, *N* = 26) and no antibiotics group (NA, *N* = 94) based on postnatal antibiotic exposure. To exclude the influence of antibiotics, the impacts of delivery mode and feeding pattern were analyzed in the remaining 94 infants from NA group, who were divided into vaginally delivered (V group, *N* = 60) and cesarean delivered (C group, *N* = 34) groups and exclusive breastfeeding (B group, *N* = 67) and mixed feeding (M group, *N* = 27) groups. In general, the baseline clinical characteristics were comparable among the different groups (Tables 1, 2). For the C group, 8.8% (3/34) were delivered by labored cesarean section, 2.9% (1/34) of pregnant woman had suffered preeclampsia and 2.9% (1/34) of pregnant woman had suffered chronic hypertension (Supplementary Table S1), which was too few for further analysis.

**Table 1:** Characteristics of infants by postnatal antibiotic exposure (*n* = 120).

	Mean ±*SD* or n (%)			^1^*P*-value
		
Characteristic	All (*N* = 120)	No antibiotics (*N* = 94)	Antibiotics (*N* = 26)	
Maternal age	32.3 ± 3.5	32.5 ± 3.6	31.6 ± 3.1	0.26
Gestational age (week ± day)	39.3 ± 1.2	39.2 ± 1.2	39.5 ± 0.8	0.45
Sampling time	49.5 ± 7.7	49.4 ± 7.8	50.2 ± 7.2	0.49
Birth weight (g)	3334.0 ± 394.2	3327.0 ± 381.4	3358.5 ± 447.0	0.96
Infant weight at 6 weeks(g)	5697.6 ± 854.3	5575.6 ± 838.5	6139.7 ± 772.5	0.01
Maternal pre-pregnancy BMI	22.8 ± 3.3	22.6 ± 3.4	23.3 ± 3.1	0.33
Gender				0.50
Male (%)	70 (58.3)	53 (56.4)	17 (65.4)	
Female (%)	50 (41.7)	41 (43.6)	9 (34.6)	
Delivery mode				0.16
Vaginally delivered	81 (67.5)	60 (63.8)	21 (81.8)	
Cesarean delivered	39 (32.5)	34 (36.2)	5 (19.2)	
Feeding pattern				0.81
Exclusively breastfed	85 (70.3)	67 (71.3)	18 (69.2)	
Mixed-fed	35 (29.7)	27 (28.7)	8 (30.8)	
Supplement of probiotics	51 (42.5)	41 (43.6)	10 (38.5)	0.66


*^*1*^*P*-value determined by Student’s *t*-test, Mann-Whitney *U*-test or Fisher’s exact test.*

**Table 2:** Characteristics of infants by delivery mode and feeding pattern (*n* = 94).

	Mean ±*SD* or n (%)			^1^*P*-value	Mean ±*SD* or n (%)		^1^*P-*value
				
Characteristic	All (*N* = 94)	Cesarean delivery (*N* = 34)	Vaginal delivery (*N* = 60)		Exclusive breast-fed (*N* = 67)	Mixed-fed (*N* = 27)	
Maternal age	32.6 ± 3.6	33.4 ± 3.3	32.1 ± 3.7	0.11	32.8 ± 3.7	31.8 ± 3.4	0.28
Gestational age (week ± day)	39.1 ± 1.2	38.9 ± 0.9	39.3 ± 1.4	0.03	39.1 ± 1.2	39.3 ± 1.4	0.30
Sampling time	49.4 ± 7.9	49.2 ± 6.9	49.5 ± 8.4	0.96	50.2 ± 8.3	47.3 ± 6.1	0.12
Birth weight (g)	3327.0 ± 381.7	3395.9 ± 417.4	3288.0 ± 357.6	0.14	3315.9 ± 392.2	3354.4 ± 359.2	0.51
Infant weight at 6 weeks (g)	5553.4 ± 840.7	5537.3 ± 980.5	5562.5 ± 758.9	0.65	5477.3 ± 868.1	5816.7 ± 716.9	0.05
Maternal pre-pregnancy BMI	22.7 ± 3.3	23.4 ± 2.8	22.2 ± 3.5	0.03	22.7 ± 3.4	22.7 ± 3.2	1
Gender				0.07			0.41
Male	53 (56.4)	15 (44.1)	38 (71.7)		36 (53.7)	17 (63.0)	
Female	41 (43.6)	19 (55.9)	22 (36.7)		31 (46.3)	10 (37.0)	
Delivery mode				–			
Vaginally delivered	60 (63.8)	–	–		43 (64.2)	17 (63.0)	0.91
Cesarean delivered	34 (36.2)	–	–		24 (35.8)	10 (37.0)	
Feeding pattern				0.91			–
Exclusively breastfed	67 (71.3)	24 (70.6)	43 (71.7)		–	–	
Mixed-fed	27 (28.7)	10 (29.4)	17 (28.3)		–	–	
Supplement of probiotics	42 (44.7)	17 (50)	25 (41.7)	0.435	34 (50.7)	8 (29.6)	0.06


*^*1*^*P-*value determined by Student’s *t*-test, Mann-Whitney *U*-test or Fisher’s exact test.*

We identified 3,367,929 bacterial sequences in 120 infant fecal samples. By evaluating the average relative bacterial abundance, we detected 8 phyla and 147 genera in all of the fecal samples. Firmicutes (44.9%) was the most abundant phylum, followed by Proteobacteria (22.7%), Actinobacteria (18.7%), and Bacteroidetes (13.2%). The top 10 abundant genera were *Bifidobacterium* (17.3%), *Clostridium sensu stricto 1* (12.8%), *Klebsiella* (10.7%), *Bacteroides* (8.7%), *Streptococcus* (8.0%), *Escherichia-Shigella* (6.2%), *Veillonella* (4.0%), *Erysipelatoclostridium* (2.2%), *Faecalibacterium* (2.0%), and *Enterococcus* (1.7%), respectively.

### The Impact of Delivery Mode and Feeding Pattern on Infant Gut Microbiota

**FIGURE 1:**
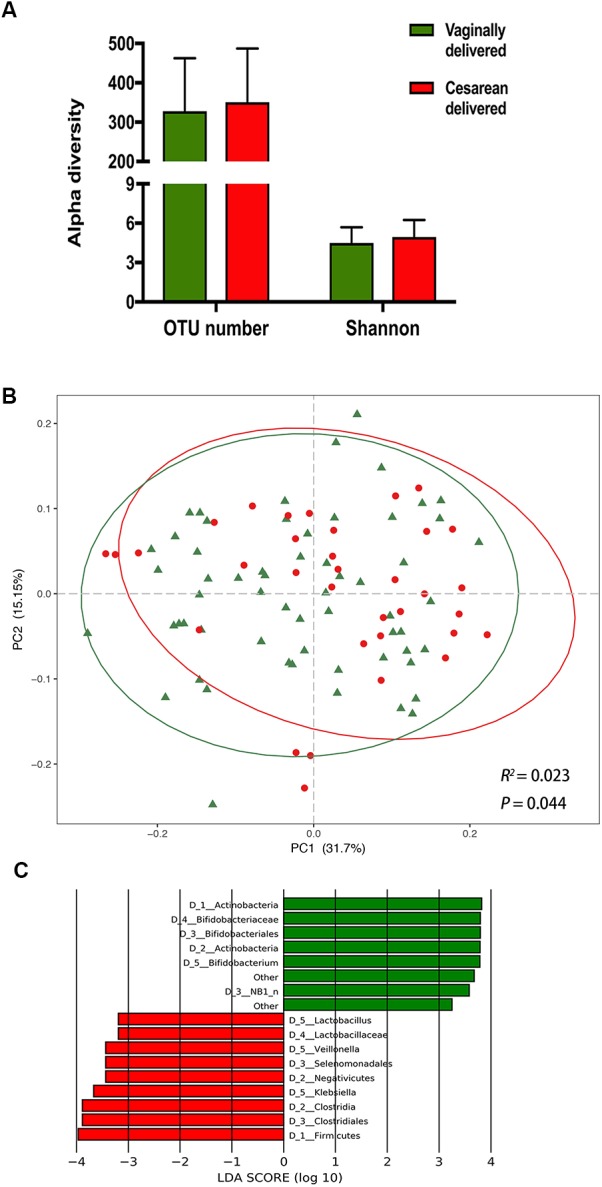
The impact of delivery mode on infant gut microbiota. **(A)** Comparison of alpha diversity between vaginally delivered and cesarean delivered infants. **(B)** Principal coordinates analysis (PCoA) based on weighted UniFrac distances is shown along the first two principal coordinate (PC) axes with Adonis *p*-value. Percentages are the percent variation explained by each PC axis. Individual samples are represented by green triangles (vaginally delivered) and red points (cesarean delivered). **(C)** Enriched taxa of different level from vaginally delivered infants with a positive linear discriminant analysis (LDA) score are shown in green; cesarean delivered infants with negative LDA score are shown in red (cut off value ≥3).

We calculated the OTU number and Shannon index to evaluate the alpha diversity of infant gut microbiome. No significant difference was found between vaginally delivered and cesarean delivered infants in either OTU number or Shannon index (Figure 1A). PCoA was applied to characterize the microbial community structures based on weighted and unweighted UniFrac distances. Delivery mode was significantly altered the microbial community structure in the weighted UniFrac analysis (Figure 1B, Adonis, *R*^2^ = 0.023, *P* = 0.044) but not in the unweighted UniFrac analysis (Supplementary Figure S1, Adonis, *R*^2^ = 0.007, *P* = 0.807). LEfSe analysis was performed to investigate the differences in the bacterial relative abundance between groups. Compared with the V group, the C group was characterized by a decrease in phylum Actinobacteria, class Actinobacteria, order Bifidobacteria and family Bifidobacteriaceae, as well as an increase in phylum Firmicutes, classes Negativicutes and Clostridia, orders Selenomonadales and Clostridiales, and family Lactobacillaceae. Four genera were significantly different: *Bifidobacterium* was enriched in the V group, and *Lactobacillus, Veillonella*, and *Klebsiella* were enriched in the C group (Figure 1C).

**FIGURE 2:**
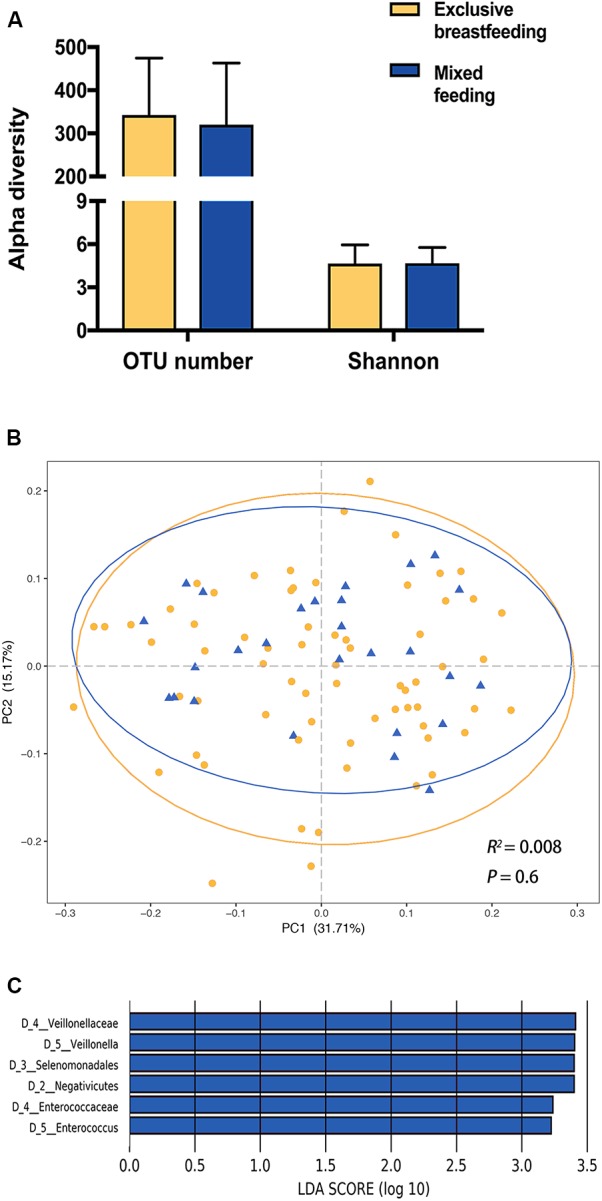
The impact of feeding pattern on the infant gut microbiota. **(A)** Comparison of alpha diversity between exclusively breastfed and mixed-fed infants. **(B)** Principal coordinates analysis (PCoA) based on weighted UniFrac distances is shown along the first two principal coordinate (PC) axes with Adonis *p*-value. Percentages are the percent variation explained by each PC axis. Individual samples are represented by yellow points (exclusively breastfed) and blue triangles (mixed-fed). **(C)** Enriched taxa of different level from mixed-fed infants with a positive linear discriminant analysis (LDA) score are shown in blue (cut off value >3).

Contrastingly, neither OTU number nor Shannon index showed significant differences between exclusively breastfed and mixed-fed infants (Figure 2A). The PCoA analysis exerted no statistically significant differences between two feeding groups, in either the weighted (Figure 2B, Adonis, *R*^2^ = 0.008, *P* = 0.6) or unweighted UniFrac analysis (Supplementary Figure S2, Adonis, *R*^2^ = 0.016, *P* = 0.15). The LEfSe analysis found that mixed-fed infants harbored more class Negativicutes, order Selenomonadales, families Veillonellaceae and Enterococcaceae and genera *Veillonella* and *Enterococcus*, compared with the exclusively breastfed infants (Figure 2C).

### Exclusive Breastfeeding Restores the Healthy Gut Microbiome in Cesarean Delivered Infants

**FIGURE 3:**
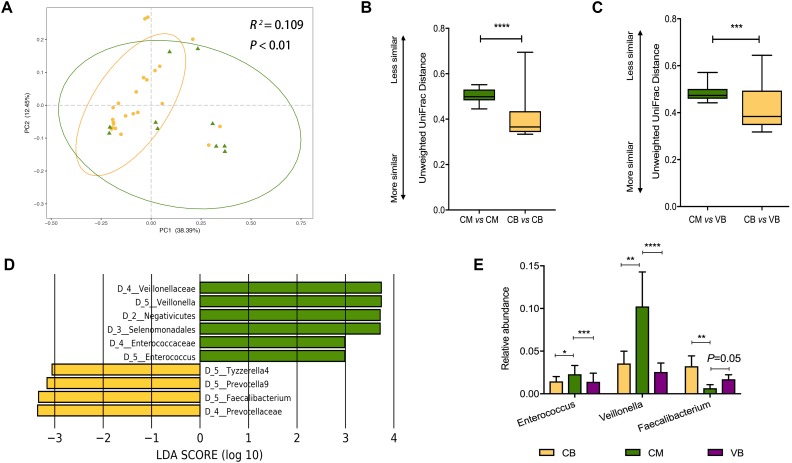
Exclusive breastfeeding significantly restores the gut microbiota of cesarean delivered infants. **(A)** Principal coordinates analysis (PCoA) based on unweighted UniFrac distances is shown along the first two principal coordinate (PC) axes with Adonis *p*-value. Percentages are the percent variation explained by each PC axis. Individual samples are represented by yellow points (CB) and green triangles (CM). Comparison of the within-groups **(B)** and between-groups **(C)** unweighted UniFrac distances of CB and CM infants. Shorter distance indicated greater similarity between microbial community composition. Significant difference was determined by Mann-Whitney *U*-test (^∗^*p* < 0.05, ^∗∗^*p* < 0.01, ^∗∗∗^*p* < 0.001, ^∗∗∗∗^*p* < 0.0001). **(D)** Enriched taxa of different level from CB infants with a positive linear discriminant analysis (LDA) score are shown in yellow; CM infants with negative LDA score are shown in green (cut off value ≥3). **(E)** Comparison of relative abundance of *Enterococcus, Veillonella*, and *Faecalibacterium* among VB, CB, and CM infants. VB, vaginally delivered and exclusive breastfed; CB, cesarean delivered and exclusive breastfed; CM, cesarean delivered and mixed-fed.

Taking the effect of delivery mode into account, the 94 infants were reorganized into four groups based on a combination of delivery mode and feeding pattern – vaginally delivered and exclusively breastfed (VB group, *N* = 43), vaginally delivered and mixed-fed (VM group, *N* = 17), cesarean delivered and exclusively breastfed (CB group, *N* = 24) and cesarean delivered and mixed-fed (CM group, *N* = 10)– to explore the effect of feeding pattern on vaginally delivered and cesarean delivered infants, respectively. Neither VB and VM groups, nor CB and CM groups have alpha diversity significant difference (Supplementary Figure S3A). For all the vaginally delivered infants (*N* = 60), feeding pattern was not significantly associated with gut microbiome composition (Supplementary Figure S3B, weighted Adonis, *R*^2^ = 0.007, *P* = 0.85; Supplementary Figure S3C, unweighted Adonis, *R*^2^ = 0.007, *P* = 0.98). As for all the cesarean delivered infants (*N* = 34), the association between feeding pattern and gut microbiome composition was statistically significant based on unweighted UniFrac distances (Figure 3A, Adonis, *R*^2^ = 0.109, *P* < 0.01), but was not statistically significant based on weighted UniFrac distances (Supplementary Figure S3D, Adonis, *R*^2^ = 0.015, *P* = 0.82). Further, we calculated the within- and between-group weighted and unweighted UniFrac distances to evaluate the microbial compositional phylogenetic similarities, and found the significantly smaller unweighted UniFrac distance within CB group than that within CM group (Figure 3B, *P* < 0.001), suggesting that CB infants shared a more unique gut microbiota than did CM infants, though the within-group weighted UniFrac distance were similar between two groups (Supplementary Figure S4A).

Our results suggested feeding pattern influenced infant microbiota in a delivery mode-dependent manner, therefore, we hypothesized that cesarean delivered infants harbor a relatively “unhealthy” gut microbiome, which could be ameliorated by exclusive breastfeeding. To test this hypothesis, we treated VB infants as a “healthy” reference, since vaginal delivery and exclusive breastfeeding are considered a natural and healthy process for initial establishment of infant gut microbiota (Azad et al., 2016; Bokulich et al., 2016), and made comparisons among VB, CB, and CM groups. We found that the unweighted UniFrac distance between VB and CB groups was significantly smaller than that between VB and CM groups (Figure 3C, *P* < 0.001), which indicated that the CB infants shared a more similar gut microbiota with VB infants than CM infants did. Still, this between-group difference was not observed in weighted UniFrac distance (Supplementary Figure S4B).

The LEfSe showed that the relative abundances of three of the top 10 predominant genera (*Enterococcus, Veillonella*, and *Faecalibacterium*) were significantly different between the CB and CM infants (Figure 3D). Regarding VB infants as a reference, we compared the relative abundances of the three genera among VB, CB, and CM infants. Compared with CM infants, we found a significant decrease in *Enterococcus* (*P* = 0.0002; *P* = 0.028) and *Veillonella* (*P* < 0.0001; *P* = 0.0036), in parallel with an increase in *Faecalibacterium* (*P* = 0.05; *P* = 0.009) in both VB and CB infants, respectively (Figure 3D). When we compared VB infants with CB infants, none of those three genera exhibited significant differences (Figure 3E). Our results suggested that CB infants shared more predominant taxa with VB infants than CM infants did.

### Postnatal Antibiotic Exposure Has a Limited Influence on Infant Gut Microbiome

**FIGURE 4:**
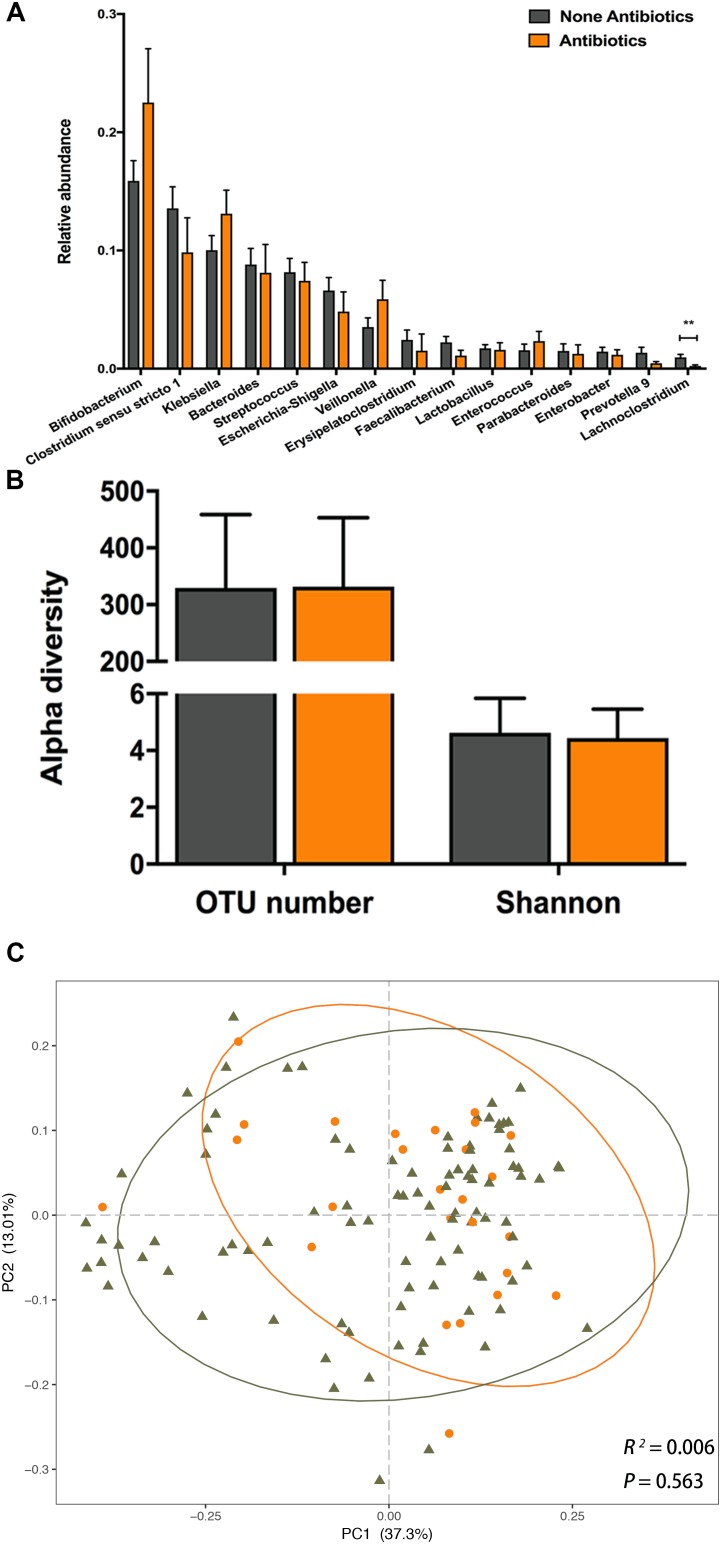
The impact of postnatal antibiotic exposure on the infant gut microbiota. **(A)** Comparison of relative abundance of the top 15 genera between antibiotic exposed and unexposed infants. Significant difference was determined by Mann-Whitney *U*-test (^∗∗^*p* < 0.01). **(B)** Comparison of alpha diversity between antibiotic exposed (orange) and unexposed (dark gray) infants. **(C)** Principal coordinates analysis (PCoA) based on weighted UniFrac distances is shown along the first two principal coordinate (PC) axes with Adonis *p*-value. Percentages are the percent variation explained by each PC axis. Individual samples are represented by orange points (antibiotic exposed) and dark gray triangles (antibiotic unexposed).

Detailed antibiotic usage information was documented from the birth to the time of sample collection (Supplementary Table S2). We compared the gut microbial profile of infants exposed and unexposed to antibiotics by 6 weeks of age. No significant differences were found in the relative abundances of the top 15 dominant taxa between the antibiotics exposed and unexposed infants (Figure 4A), except for genus *Lachnoclostridium*, which was significantly diminished in exposed infants (0.23 ± 0.01% vs. 0.96 ± 0.05%, *P* = 0.006). With detailed antibiotic usage information, we further explored the correlation between the relative abundance of *Lachnoclostridium* and antibiotic cumulative dosage, as well as interval time (the time from antibiotic exposure to sample collection). However, no significant relationship was observed between the relative abundance and cumulative dosage (*r* = -0.09, *P* = 0.63), or interval time (*r* = -0.10, *P* = 0.61).

The OTU number and Shannon index were not significantly different between the NA and A groups (Figure 4B). Furthermore, we observed no significant difference in PCoA based on weighted or unweighted UniFrac distances between the NA and A groups (Figure 4C, Adonis, *R*^2^ = 0.006, *P* = 0.563; Supplementary Figure S5, Adonis, *R*^2^ = 0.004, *P* = 0.927). In LEfSe analysis between two groups, we did not find any difference in the relative abundances of gut microbiota. These results suggested that neither gut microbial composition nor structure of infants aged 6 weeks was influenced by the postnatal antibiotic exposure.

## Discussion

We performed a cross-sectional study with 120 infants to evaluate the impacts of delivery mode, feeding pattern and postnatal antibiotic exposure on their intestinal microbiomes and to identify the restorative effect of exclusive breastfeeding on gut microbiota of cesarean delivered infants. Despite geographical and ethnic variations, the gut microbial compositions of these 120 infants were consistent with those of previous studies (Martin et al., 2016; Hill et al., 2017; Nagpal et al., 2017), with a predominance of phyla Firmicutes, Proteobacteria, Actinobacteria and Bacteroidetes, in parallel with genera *Bifidobacterium, Clostridium* and *Bacteroides* by the age of 6 weeks.

We identified a significant impact of delivery mode on establishment of infant gut microbial composition and structure. The alteration of the microbial structure was not observed in the unweighted UniFrac analysis, but was evident in the weighted UniFrac analysis, which indicated that the phylogeny of gut microbiota showed no statistical significance, whereas the abundance of gut microbiota was significantly differed between vaginally delivered and cesarean delivered infants by the age of 6 weeks.

In our study, *Bifidobacterium* was enriched in vaginally delivered infants at different OTU levels when compared with cesarean delivered ones. The same result was also observed in previous studies (Hesla et al., 2014; Dogra et al., 2015), which found that the low abundance of *Bifidobacterium* following cesarean section was evident within 3 weeks of age, but was no longer detectable at 2 months of life (Hesla et al., 2014). In a recent study, a 24 age-discriminatory taxon model including *Bifidobacterium* was used to predict the microbiota maturity of infants who were suffered from the acute malnutrition, which indicated that the *Bifidobacterium* might play an important role in the establishment and maturation of infant gut microbiome (Subramanian et al., 2014). Additionally, the *Bifidobacterium* is known for its health promoting properties and ability to stimulate the immune response (Turroni et al., 2012; Quagliariello et al., 2016). A recent study also found that delayed colonization of *Bifidobacterium* in infants delivered by cesarean section was linked with adiposity at 18 months of life (Dogra et al., 2015). In contrast, genera *Klebsiella* and *Veillonella* were significantly overrepresented in cesarean delivered infants. *Klebsiella* is a common opportunity pathogenic bacterium in hospitals and usually is identified in cesarean delivered infants (Stokholm et al., 2016) who are more often admitted to the neonatal ward, whereas the *Veillonella* is associated with the penetrating complications of children Crohn’s disease (Kugathasan et al., 2017). Therefore, our findings suggested that the gut microbial abnormalities and delayed maturation were associated with cesarean delivery, which was consistent with previous studies in humans (Hesla et al., 2014; Dogra et al., 2015; Bokulich et al., 2016) and mice (Cox et al., 2014; Martinez et al., 2017).

Regarding to feeding pattern, we did not find any differences in alpha diversity or community structure between exclusively breastfed and mixed-fed infants, which might due to the rough classification method. For the breastfed infants, 27.7% of their gut bacteria are acquired from maternal breast milk, and gut microbial changes are associated with the proportion of daily breast milk intake in a dose-dependent manner (Pannaraj et al., 2017). However, all of our subjects were fed with breast milk in various proportions, lacking of quantifiable data of daily milk intake, which might eliminate the microbial differences between exclusive breastfeeding and mixed feeding. Future studies are warranted to explore the relationship between proportion of daily breast milk intake and gut microbiota, specifically the impact of breast milk composition on gut microbiota.

An important finding of our study was that compared with the CM infants, the CB infants shared a more similar gut microbial composition and community structure with the VB infants at 6 weeks of age. Coincidentally, Hill et al. (2017) recently found that cesarean delivered infants gradually developed a gut microbiota that closely resembled that of vaginally delivered infants by breastfeeding from birth to 24 weeks of life based on a comparison of the relative abundances of bacteria at the phylum level. Our study drew the same conclusion by comparing the relative abundances of bacteria at the genus level, which mutually complemented Hill’s work to a certain extent. As far as we know, mixed feeding is relatively rare in many parts of world, and previous studies tended to make comparisons of breastfeeding versus formula feeding (Lee et al., 2015; Bokulich et al., 2016). In this study, we explored the impact of exclusive breastfeeding and mixed feeding on infant gut microbiome, and found that the cesarean-born infants could acquire a gut microbial community resembled the vaginally delivered infants by exclusively breastfed. Future studies are needed to investigate the relationship between different feeding pattern and gut microbiome profiles, as well as healthy outcomes at different stages of the life course.

We considered VB infants as a healthy and natural reference because breast milk helps shape the healthy establishment of infant gut microbiome through its beneficial components (Le Doare et al., 2018), such as immunoglobulin (Ig) A, cytokines and lactoferrin, which protected against pathogenic bacteria and viruses (Lonnerdal, 2003). As an important component of breast milk, human milk oligosaccharides (HMOs) served as an energy source for a limited number of gut bacteria, including Bifidobacteria and Bacteroidetes, but not for pathogenic bacteria (Chong et al., 2018). As observed *in vitro* (Marcobal et al., 2010), HMOs cannot be carbon sources for the growth of the *Veillonella* or *Enterococcus* strains, which may explain the differences in the relative abundance of *Veillonella* and *Enterococcus* between exclusively breastfed and mixed-fed infants. Our finding indirectly illustrated that exclusive breastfeeding partially restored the disturbance of gut microbiota in cesarean delivered infants and that the introduction of formula milk might reduce the benefits of exclusive breastfeeding. This finding also provided the support to the tenets of the World Health Organization’s Baby Friendly Hospital Initiative, which widely advocates that exclusive breastfeeding should start at birth in the hospital and continue at least to 6 months of life^2^.

Regarding postnatal antibiotic exposure, the relative abundance of *Lachnoclostridium* (a genus of family Lachnospiraceae) was significantly decreased in the antibiotic exposed infants. The depletion of Lachnospiraceae along with other specific OTUs may delay intestinal microbial community development in antibiotic-exposed infants; however, this effect is only evident from 6 to 12 months and gradually disappears thereafter (Bokulich et al., 2016). Additionally, the proportion of Lachnospiraceae decreases in healthy adults after administration of amoxicillin-clavulanic acid for 1 week and recovers to the baseline level 1 week after cessation of antibiotic exposure (MacPherson et al., 2018). In our study, most infants (22 of 26) were orally administered cefaclor (0.12 g day^-1^) in the first several days (day 1 to day 4) of life. Expect for the *Lachnoclostridium*, postnatal antibiotic exposure did not significantly affect the relative abundances of the predominant genera by 6 weeks of age. This inconsistency may due to the variations in the antibiotic type, dosage and duration. With the prudent medical option, a low dose and short course of antibiotic treatment may limit the disturbed impact of postnatal antibiotic exposure on initial establishment of gut microbiome.

Our study was limited by the single cross-sectional data, lacking of consecutive sampling during early infancy and the follow-up period was too short to observe the dynamic effects of aforementioned factors on infant gut microbiome. Future studies are needed to determine the longitudinal and dynamic impacts of the aforementioned factors on initial establishment of infant gut microbiota.

## Conclusion

This study indicates an independent impact of delivery mode on infant gut microbiota by the 6 weeks of age with markedly different bacterial structures and compositions, whereas no significant difference was found when the feeding pattern or postnatal antibiotic exposure was evaluated separately. The perturbation of gut microbiome establishment caused by the cesarean delivery is partially restored by exclusive breastfeeding, resulting in a gut microbial composition and community structure similar to those of vaginally delivered and exclusive breastfeeding infants, who are considered to be a healthy and natural reference. Antibiotic exposure in the first days of life seems to have no influence on infant gut microbiome by the age of 6 weeks. Our results emphasize the benefits of exclusive breastfeeding on the healthy gut microbial establishment, especially for the cesarean delivered infants.

## Data Availability

The datasets used and/or analyzed during the current study are available from the Sequence Read Archive (SRA), under BioProject PRJNA489137.

## Author Contributions

YL, SQ, BZ, JM, and HY conceived the study design. YL, SQ, YS, YF, and SW were responsible for the recruitment and collection of samples. NL, YX, and FL were responsible for the laboratory assays. YL and SQ performed the data analysis, and YL completed the initial manuscript. BZ, JM, and HY revised the manuscript. All the authors read and approved the final version of the manuscript.

## Conflict of Interest Statement

The authors declare that the research was conducted in the absence of any commercial or financial relationships that could be construed as a potential conflict of interest.

## Footnotes

1 https://support.illumina.com/downloads/16s_metagenomic_sequencing_library_preparation.html

2 http://www.who.int/nutrition/topics/bfhi/en/
