# Supplementary material for: The Perturbation of Infant Gut Microbiota Caused by Cesarean Delivery Is Partially Restored by Exclusive Breastfeeding
Source: Front Microbiol. 2019 Mar 26;10:598. doi: 10.3389/fmicb.2019.00598 (PMC6443713; doi:10.3389/fmicb.2019.00598)
Supplement: Supplementary file 1 [file Data_Sheet_1.docx]

**Supplement Figures: S1-S5**


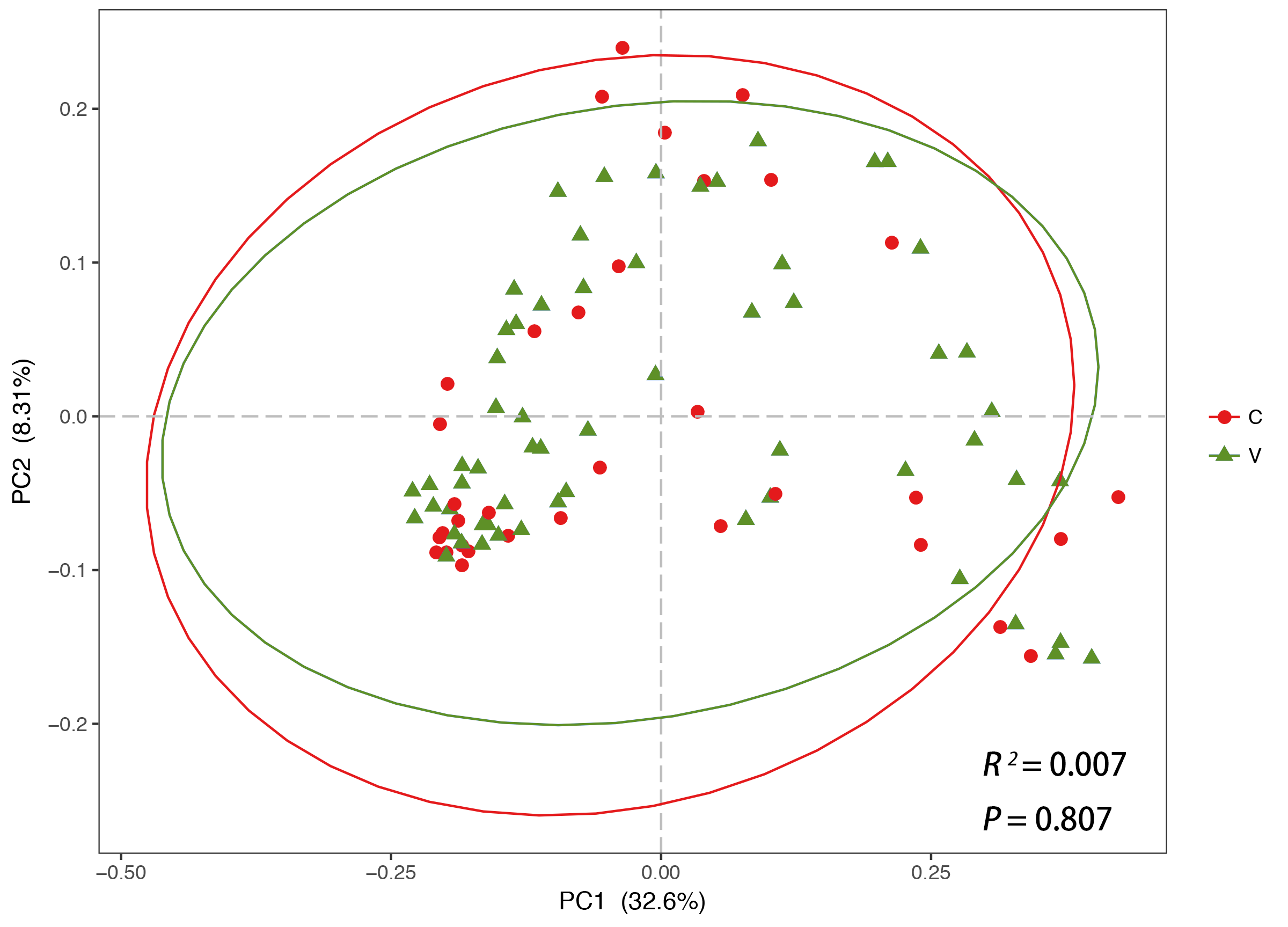


**Figure S1** Principal coordinates analysis (PCoA) based on unweighted UniFrac distances is shown along the first two principal coordinate (PC) axes with Adonis *p* value. Percentages are the percent variation explained by each PC axis. V, vaginally delivered; C, cesarean delivered.


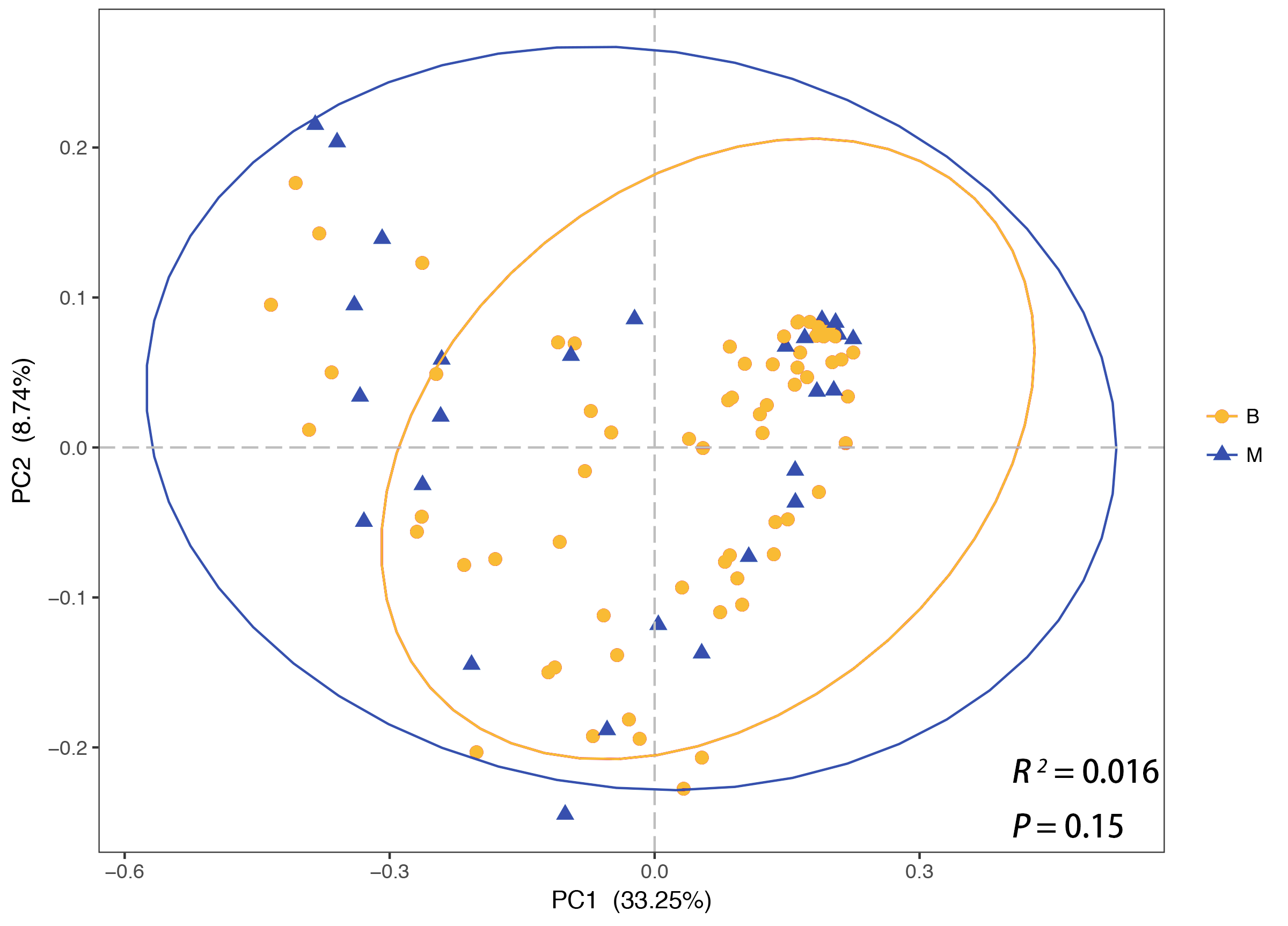


**Figure S2** Principal coordinates analysis (PCoA) based on unweighted UniFrac distances is shown along the first two principal coordinate (PC) axes with Adonis *p* value. Percentages are the percent variation explained by each PC axis. B, exclusively breastfed; M, mixed-fed.


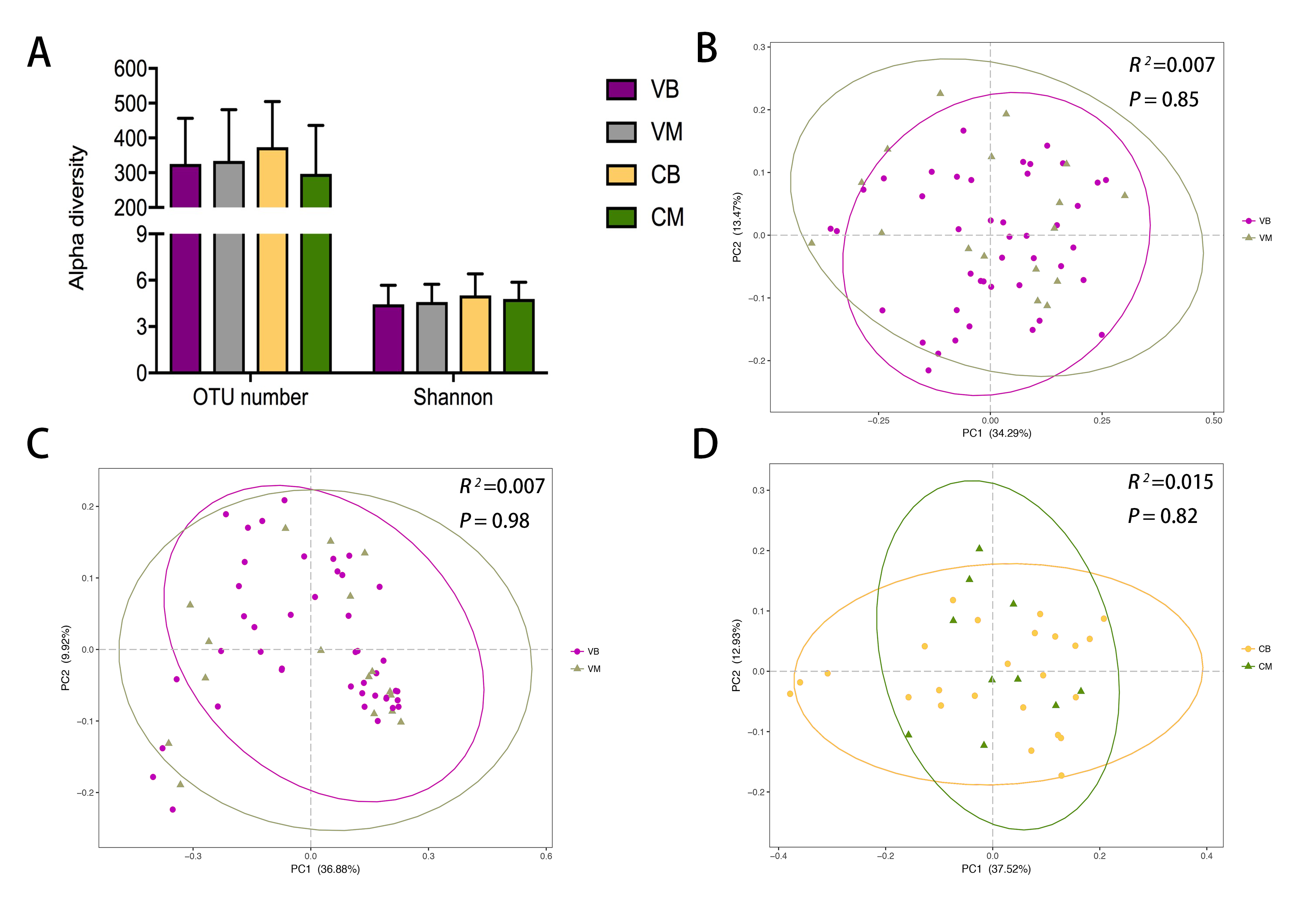


**Figure S3** (A) Comparison of alpha diversity between VB and VM, CB and CM infants. Principal coordinates analysis (PCoA) based on weighted (B and D) / unweighted (C) UniFrac distances is shown along the first two principal coordinate (PC) axes with Adonis *p* value. Percentages are the percent variation explained by each PC axis. VB, vaginally delivered and exclusive breastfed; CB, cesarean delivered and exclusive breastfed; CM, cesarean delivered and mixed-fed.


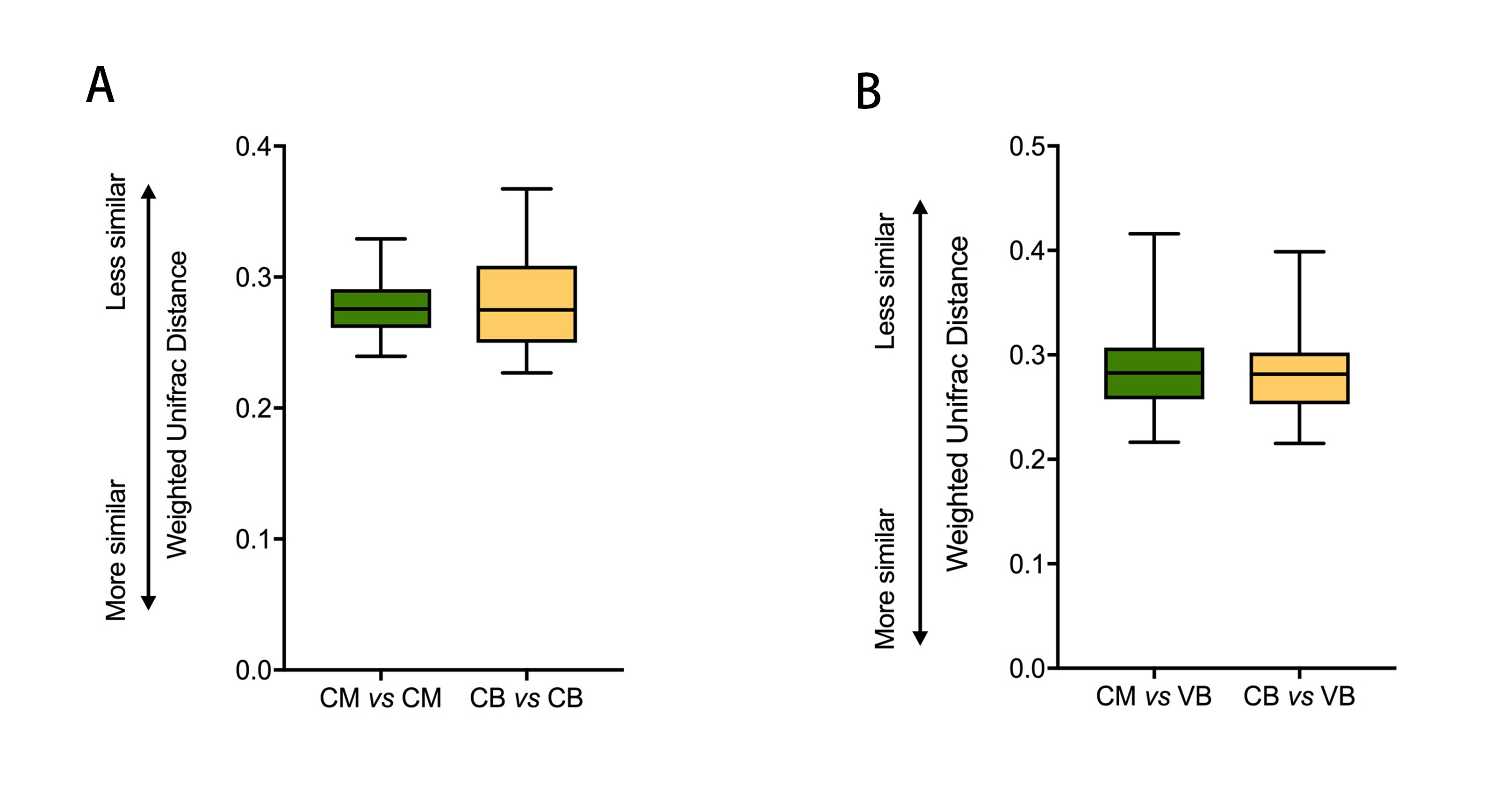


**Figure S4** Comparison of the within-groups (A) and between-groups (B) weighted UniFrac distances of CB and CM infants. Shorter distance indicated greater similarity between microbial community composition. Significant difference was determined by Mann-Whitney U test.


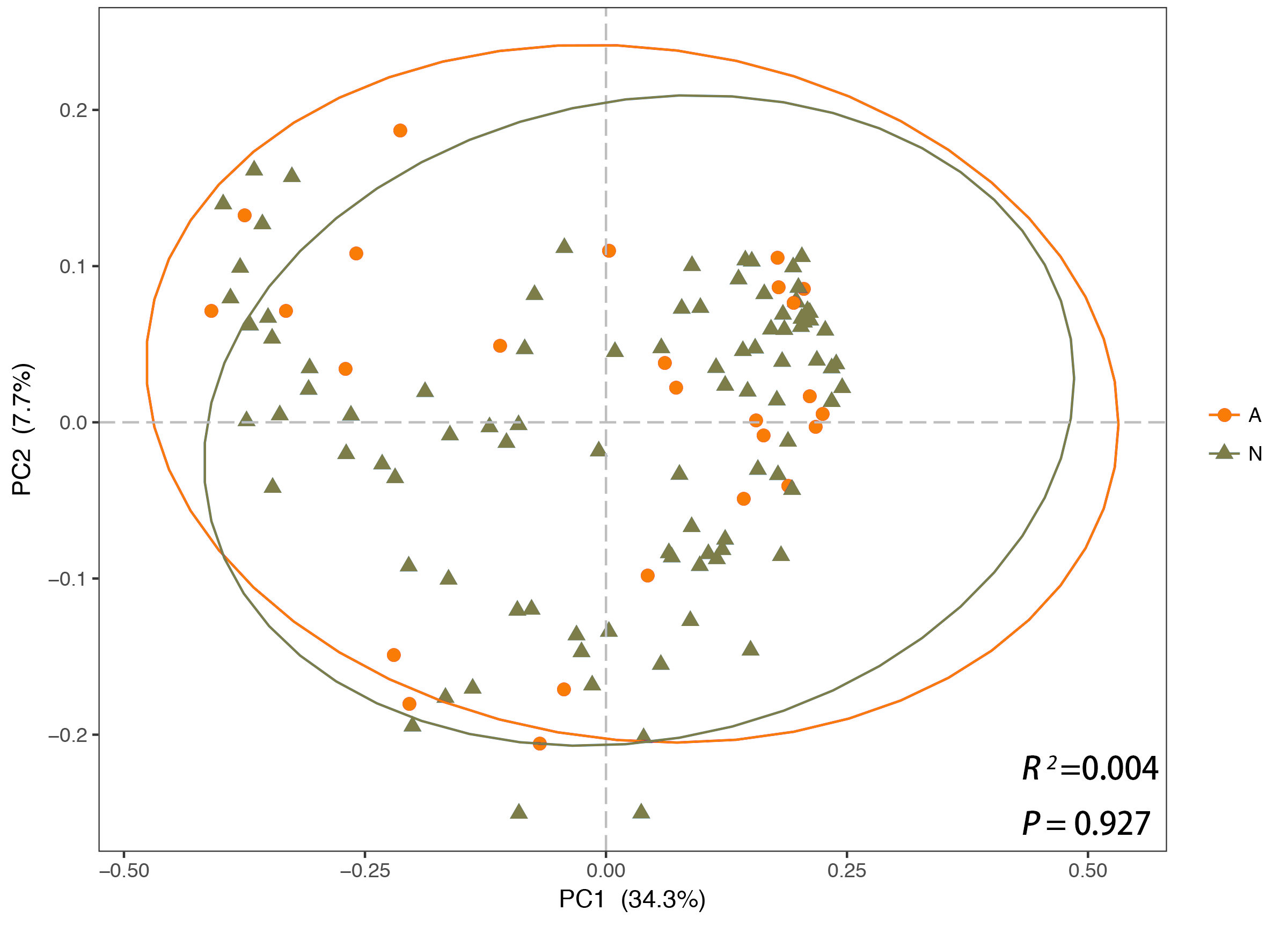


**Figure S5** Principal coordinates analysis (PCoA) based on unweighted UniFrac distances is shown along the first two principal coordinate (PC) axes with Adonis *p* value. Percentages are the percent variation explained by each PC axis. A, antibiotic exposed; N, antibiotic unexposed.
